# Recurrent Chromosome 22 Deletions in Osteoblastoma Affect Inhibitors of the Wnt/Beta-Catenin Signaling Pathway

**DOI:** 10.1371/journal.pone.0080725

**Published:** 2013-11-13

**Authors:** Karolin H. Nord, Jenny Nilsson, Elsa Arbajian, Fredrik Vult von Steyern, Otte Brosjö, Anne-Marie Cleton-Jansen, Karoly Szuhai, Pancras C. W. Hogendoorn

**Affiliations:** 1 Department of Clinical Genetics, University and Regional Laboratories, Skåne University Hospital, Lund University, Lund, Sweden; 2 Department of Orthopedics, Skåne University Hospital, Lund, Sweden; 3 Department of Orthopedics, Karolinska Hospital, Stockholm, Sweden; 4 Department of Pathology, Leiden University Medical Center, Leiden, The Netherlands; 5 Department of Molecular Cell Biology, Leiden University Medical Center, Leiden, The Netherlands; National Cancer Center, Japan

## Abstract

Osteoblastoma is a bone forming tumor with histological features highly similar to osteoid osteoma; the discrimination between the tumor types is based on size and growth pattern. The vast majority of osteoblastomas are benign but there is a group of so-called aggressive osteoblastomas that can be diagnostically challenging at the histopathological level. The genetic aberrations required for osteoblastoma development are not known and no genetic difference between conventional and aggressive osteoblastoma has been reported. In order to identify recurrent genomic aberrations of importance for tumor development we applied cytogenetic and/or SNP array analyses on nine conventional and two aggressive osteoblastomas. The conventional osteoblastomas showed few or no acquired genetic aberrations while the aggressive tumors displayed heavily rearranged genomes. In one of the aggressive osteoblastomas, three neighboring regions in chromosome band 22q12 were homozygously deleted. Hemizygous deletions of these regions were found in two additional cases, one aggressive and one conventional. In total, 10 genes were recurrently and homozygously lost in osteoblastoma. Four of them are functionally involved in regulating osteogenesis and/or tumorigenesis. *MN1* and *NF2* have previously been implicated in the development of leukemia and solid tumors, and *ZNRF3* and *KREMEN1* are inhibitors of the Wnt/beta-catenin signaling pathway. In line with deletions of the latter two genes, high beta-catenin protein expression has previously been reported in osteoblastoma and aberrations affecting the Wnt/beta-catenin pathway have been found in other bone lesions, including osteoma and osteosarcoma.

## Introduction

Osteoblastoma is a bone forming tumor that is usually located in the medullary cavity of the bone [1]. The disease can affect patients at any age but has a predilection for males in their teens and young adulthood. At the cellular level, the tumor is identical to osteoid osteoma; both tumor types show rich vascularization, irregular osteoid with osteoblasts and often osteoclast-type multinucleated giant cells. Differentiation between the two tumor types is based on size [2]. Osteoid osteoma has a limited growth potential and seldom exceeds 1 cm in largest diameter. In contrast, lesions larger than 2 cm are not considered to have a restricted growth potential and are referred to as osteoblastomas (also known as giant osteoid osteomas). Osteoblastoma typically shows a non-infiltrative growth pattern and when resected with free margins recurrences are uncommon. The treatment is therefore based on surgery alone and the prognosis is excellent [1]. However, there is a group of intra-osseous osteoblastic tumors that can be diagnostically challenging at the histopathological level [3]. These tumors have been referred to as aggressive, epithelioid or malignant osteoblastoma. Currently, they are considered within the morphological spectrum of osteoblastoma [1], and have the same clinical behavior. A very rare subtype of osteosarcoma exists, so-called osteoblastoma-like osteosarcoma, which shares some morphological features with osteoblastoma, but clinically behaves like conventional high-grade osteosarcoma [4]. Osteosarcomas, including these rare variants, generally present highly complex karyotypes with multiple aberrations [5]. Low-grade central osteosarcomas do not pose a histological differential diagnosis and are characterized at the genetic level by frequent gains of *MDM2*. In osteoblastoma the genetic findings are heterogeneous ranging from single balanced structural rearrangements to multiple and complex changes. No recurrent, tumor-associated aberration has been described. 

In the present study, we have applied cytogenetic and single nucleotide polymorphism (SNP) array analyses on osteoblastomas in order to identify recurrent genomic aberrations of importance for tumor development. We have also re-analyzed previously published global gene expression data on osteoblastoma [6], in order to evaluate the possible impact of genomic alterations. 

## Materials and Methods

### Ethics statement

All samples were obtained after informed written consent from patients or from patient’s parents. The study was approved by the Regional Ethics Committee of Lund University.

### Patient information and tumor material

Tumor material was available from 11 osteoblastoma patients, treated at the Leiden University Medical Center, Leiden, The Netherlands, the Skåne University Hospital, Lund, Sweden and the Karolinska Hospital, Stockholm, Sweden. The age of the patients ranged from 10-44 years and the majority (9 of 11) of patients were male. Two of the tumors were classified as epithelioid or aggressive and the remaining cases were conventional osteoblastomas. Three of the tumors recurred and in the remaining patients there was no evidence of disease 25-253 months after diagnosis. Detailed patient information can be found in Table 1. 

**Table 1:** Clinical and cytogenetic features of eleven osteoblastomas.

**Case** ^a^	**Age/Sex**	**Location**	**Follow-up** ^b^	**Karyotype** ^c^	**SNP array analysis**
1^d^	35/M	Skull	R 21, 53, NED 60	43,X,-Y,der(1)t(1;?22)(p3?;q?),add(2)(q3?),-6,del(8)(p12),der(9)t(9;17)(p12;q11),-12,der(14)t(?1;14)(?;q3?),-17,der(19)t(19;22)t(1;22)t(12;22)t(12;17),der(22)t(1;22)(?;q1?)[23]/46,XY[7]	Aberrant
2^e^ 13848:85	23/M	Proximal humerus	NED 168	44,X,-Y,add(1)(p34),del(1)(q21),del(2)(p21p23),+3,del(3)(p21)x2,del(6)(q15),der(6)t(6;13)(q27;q12),+10,der(10)t(8;10)(q11;q26)x2,del(12)(p11),-13,+der(15)t(1;15)(q21;p13)ins(1;?)(q32;?),+16,-17,add(17)(q11),-18,-22,-22[16]/43,idem,-15[3]	Aberrant
3	14/M	Vertebra	NED 48	44-45,XY,-22[cp 43]/46,XY[25]	No
4	17/F	Proximal femur	NED 25	48,XX,+2mar[9]/46,XX[17]	No
5	20/M	Proximal humerus	R 18, NED 21	46,XY,add(18)(q2?3)[7]/46,XY[17]	No
6	16/M	Proximal femur	NED 43	46,XY[25]	Normal
7	10/F	Sacrum	R 9, NED 18	46,XX[25]	Normal
8	37/M	Os ilium	NED 253	46,XY[25]	Normal
9	44/M	Vertebra	NED 156	No	Normal
10	42/M	Vertebra	NED 36	46,XY[18]	Normal
11	30/M	Aceta-bulum	NED156	No	Normal

a Previously published karyotypes are indicated by their reference and case numbers in the Mitelman Database of Chromosome Aberrations and Gene Fusions in Cancer (http://cgap.nci.nih.gov/Chromosomes/Mitelman).

b Follow-up time given in months. R = recurrence; NED = no evidence of disease.

c Karyotypes in cases 1 and 10 are based on data from COBRA FISH analysis.

d Epithelioid osteoblastoma.

e Aggressive osteoblastoma.

### Cytogenetic analyses

Chromosome banding and COBRA fluorescence in situ hybridization analyses were performed at the Departments of Clinical Genetics, Lund and Molecular Cell Biology, Leiden, and karyotypes were described according to the recommendations in ISCN 2009 [7]. Chromosome preparations were prepared as described [8]. 

### Genomic copy number and loss of heterozygosity analyses

SNP array analysis was used for combined DNA copy number and loss of heterozygosity investigation. In eight of the cases (cases 1, 2 and 6-11), fresh frozen tumor biopsies were available. DNA was extracted according to standard procedures [9], and hybridized onto Illumina Human Omni-Quad BeadChips, containing more than 1 million reporters, following protocols supplied by the manufacturer (Illumina, San Diego, CA). Data analysis was performed using the GenomeStudio software (Illumina), detecting imbalances by visual inspection. Constitutional copy number variations were excluded through comparison with the Database of Genomic Variants, http://projects.tcag.ca/variation/ [10]. 

### Global gene expression analyses

Global gene expression analyses was previously performed on osteoblastomas and osteosarcoma as well as the putative progenitor cells of the tumors, i.e., mesenchymal stem cells (MSC) and the same MSC differentiated into osteoblasts [6]. In brief, RNA was extracted from frozen tissue sections, labeled and hybridized onto Hu133A GeneChip Arrays according to the manufacturer’s protocol (Affymetrix, Santa Clara, CA). Gene expression data were normalized, background-corrected, and summarized by using the Robust Multichip Analysis algorithm implemented in the Expression Console version 1.1 software (Affymetrix). Correlation-based principal component analysis and hierarchical clustering analysis were performed using the Qlucore Omics Explorer version 2.3 (Qlucore AB, Lund, Sweden). Differences between tumor groups in log_2_ transformed expression data were calculated using a *t*-test, and corrections for multiple testing were based on the Benjamini-Hochberg method (Qlucore AB). Genes with *p* < 0.001 and a false discovery rate (FDR) < 0.01 were considered significantly altered.

### Genomic sequencing of candidate target genes

Sanger sequencing was used to screen the coding regions of *MN1, ZNRF3, KREMEN1* and *NF2* for mutations. PCR primers and protocols for all four genes are available in Table S1.

## Results

### Recurrent deletions affect chromosome 22 in osteoblastoma

**Figure 1:**
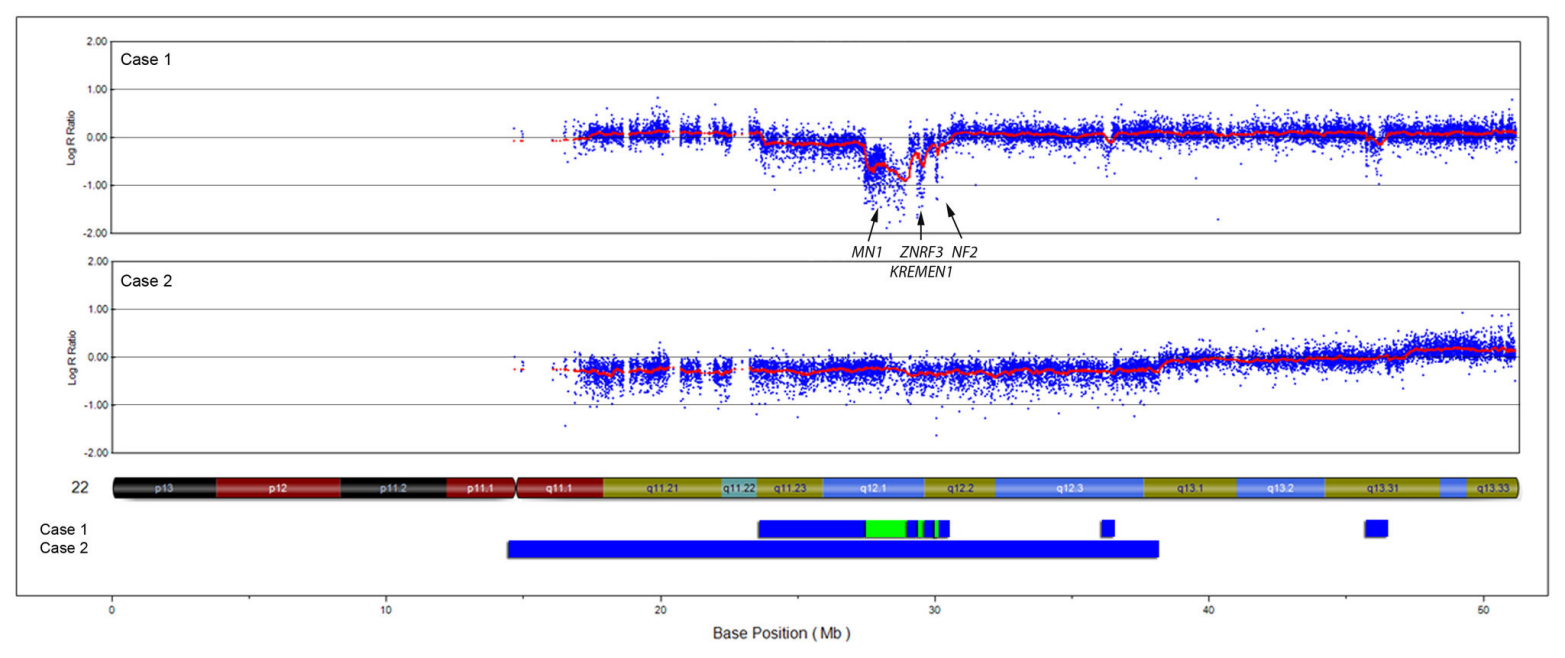
Recurrent deletions in osteoblastoma affect chromosome 22. By SNP array analyses, hemi-and homozygous deletions affecting chromosome 22 were found in cases 1 and 2. The upper parts of the figure display SNP array plots of the respective case and the lower part show a summary of the losses where blue and green bars represent hemi- and homozygous deletions, respectively. The locations of four homozygously lost genes implicated in bone formation and/or tumorigenesis are shown.

Cases 1 and 2 were diagnosed as epithelioid and aggressive osteoblastomas, respectively. In both cases, chromosome banding and COBRA fluorescent in situ hybridization analyses revealed near-diploid karyotypes with multiple and complex aberrations including deletions, gains, translocations and insertions (Table 1). In both cases, SNP array analyses showed acquired copy number alterations that were in line with the complex karyotypes. SNP array analysis of case 1 showed alterations in chromosomes 1, 2, 6, 8, 9, 12, 17 and 22, including homozygous deletions in chromosome 22 (Figure 1, Table 2). These deletions affected three distinct regions between 0.06-1.5 Mb in size and in total 10 genes, including *MN1, ZNRF3, KREMEN1* and *NF2*. In case 2, SNP array analysis showed copy number aberrations affecting chromosomes 1, 2, 3, 6, 8, 10, 16, 17, 18 and 22 (Table 2). However, loss of both copies of chromosome 22, which was found by chromosome banding, could not be corroborated by SNP array analysis. This is in line with the fact that nullisomy is a very rare finding, even at G-banding analysis [5], and that such losses most likely result in cell death. Instead, a hemizygous deletion affecting major parts of chromosome 22 was found by SNP arrays (Figure 1, Table 2). This deletion was overlapping with hemi- and homozygous deletions detected in case 1 (Figure 1). Two additional recurrent aberrations were found by SNP arrays. In chromosome 1, five small deletions (<1 Mb) affecting case 1 overlapped with a 42 Mb deletion in case 2. Chromosome 6 was deleted in case 1 and the long arm of this chromosome was lost in case 2. 

**Table 2:** Acquired DNA copy number aberrations detected by SNP arrays in two osteoblastomas.

**Case**	**Chromosome band**	**Base pair position (Mb)^a^**	**Aberration**	**Gene**
1	1p35	29.616-30.277	Deletion	
1	1p35	30.479-30.597	Deletion	
1	1p35	30.899-31.179	Deletion	
1	1p34	39.887-39.967	Deletion	
1	1p34	41.552-41.615	Deletion	
1	1p34	45.067-45.139	Deletion	
1	1p34	45.853-45.962	Deletion	
1	2p12	80.608-82.511	Gain	
1	Chromosome 6	0.090-170.919	Deletion	
1	8p21-p23	0.165-28.624	Deletion	
1	9p11-p24	0.047-47.203	Deletion	
1	12p11-p13	0.148-34.856	Deletion	
1	12q12-q13	38.922-52.786	Deletion	
1	12q13	55.311-56.366	Deletion	
1	12q24	124.643-133.779	Deletion	
1	17p11	17.271-21.528	Deletion	
1	22q11-q12	23.704-27.427	Deletion	
1	22q12	27.458-28.964	Homozygous deletion	*MN1, PITPNB, MIR3199-1 MIR3199-2, TTC28*
1	22q12	29.055-29.319	Deletion	
1	22q12	29.361-29.607	Homozygous deletion	*ZNRF3, C22orf31, KREMEN1, EMID1*
1	22q12	29.835-30.031	Deletion	
1	22q12	30.048-30.111	Homozygous deletion	*NF2*
1	22q12	30.112-30.163	Deletion	
1	22q12	30.391-30.438	Deletion	
1	22q12	36.162-36.444	Deletion	
1	22q13	45.711-46.280	Deletion	
2	1p34-p36	0.082-42.085	Deletion	
2	2p23-p25	0.015-25.967	Deletion	
2	3p11-p21	46.961-90.494	Deletion	
2	3q11-q29	93.537-197.896	Gain	
2	6q11-q27	61.885-170.919	Deletion	
2	8q11-q24	46.846-146.293	Gain	
2	Chromosome 10	0.082-135.523	Gain	
2	Chromosome 14	18.398-107.288	Copy number neutral loss of heterozygosity	
2	Chromosome 16	0.087-90.170	Gain	
2	17p11-p13	0.007-17.179	Deletion	
2	17p11	17.231-22.261	Gain	
2	17q11	25.264-27.505	Deletion	
2	Chromosome 18	0.028-78.015	Deletion	
2	22p11-q13	14.676-38.206	Deletion	
2	22q13	47.241-51.667	Gain	

a Human Feb. 2009 (GRCh37/hg19) Assembly

Three additional cases displayed chromosome alterations by cytogenetic analysis (cases 3-5; Table 1). All three cases were conventional osteoblastomas and they showed simple karyotypes with few alterations: loss of chromosome 22 (case 3), two supernumerary marker chromosomes (case 4) and addition of material on chromosome 18 (case 5). 

In summary, five out of eleven cases showed acquired DNA copy number alterations using chromosome banding and/or SNP array analyses; three of these showed homo- and/or hemizygous deletions in chromosome 22 (Tables 1 and 2). Four cases showed normal karyotypes, which could be attributed to the growth normal fibroblast. 

### Gene expression analysis supports active Wnt/beta-catenin signaling in osteoblastoma

**Figure 2:**
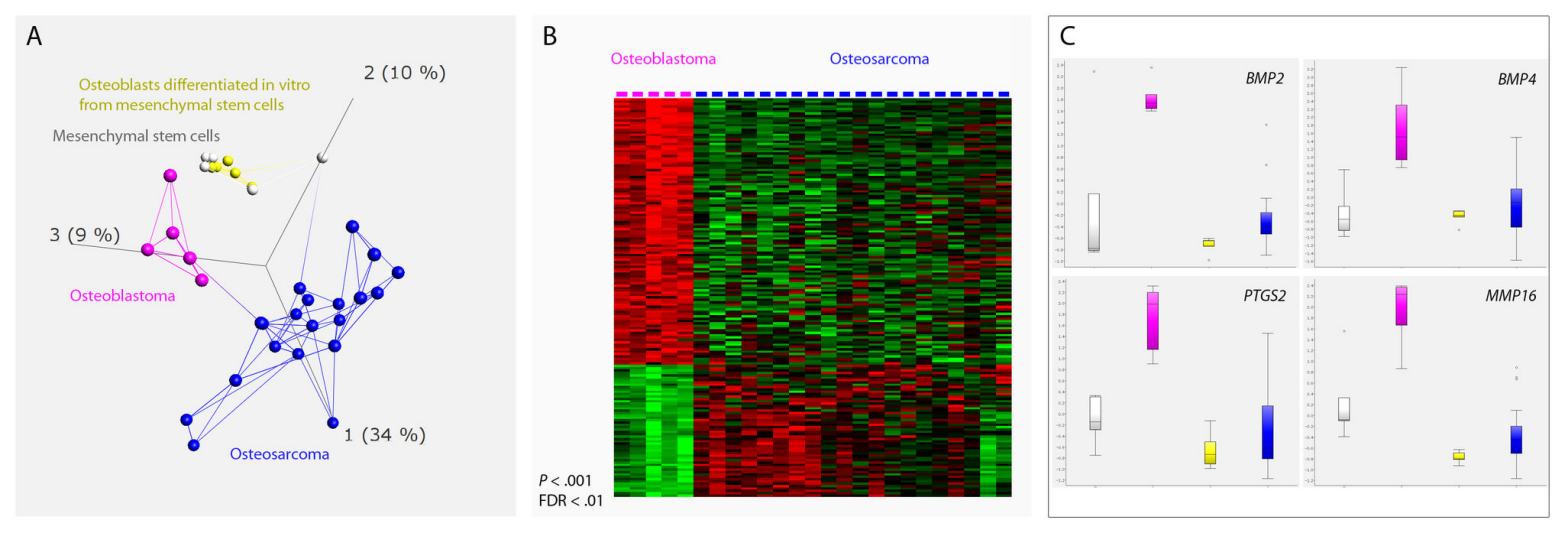
Gene expression signature of osteoblastoma. (A) Unsupervised principal component analysis based on the expression of the 1297 most variable genes (σ/σ_max_ = 0.3) shows that the five osteoblastomas form a group that has an expression profile separate from the osteosarcomas, mesenchymal stem cells, and osteoblasts differentiated in vitro from mesenchymal stem cells. The first three principal components, representing 34%, 10%, and 9% of the variance, are displayed. Lines connect the three nearest neighbors. By subsequently comparing osteoblastoma and osteosarcoma, 140 genes showed a significantly different expression (*p* < 0.001, FDR < 0.01; Tables S2 and S3). (B) The differentially expressed genes are displayed in a heat map. Genes with high and low expression values are labeled in red and green, respectively. (C) Many of the highly expressed genes in osteoblastoma are known to be involved in bone metabolism and at least four of them are induced by the Wnt/beta-catenin signaling pathway; *BMP2, BMP4, PTGS2* and *MMP16*. Boxes range from the 25^th^ to the 75^th^ percentile. The box whiskers are set at the lowest data point value still within 1.5 times the box range of the lower box limit, and at the highest data point value still within 1.5 times the box range of the upper box limit. The median is displayed as a dotted band. Outliers are defined as data point values falling outside of the box whisker limits.

By unsupervised principal component analysis, osteoblastoma displayed a distinct global gene expression signature (Figure 2). In total, 140 genes showed a significantly different expression between osteoblastoma and osteosarcoma (*p* < 0.001, FDR < 0.01; Figure 2, Tables S2 and S3). Many of the genes (>40) with a high expression level in osteoblastoma were related to bone metabolism. At least four of these - *BMP2, BMP4, PTGS2* and *MMP16* - are known to be induced by the canonical Wnt signaling pathway that controls beta-catenin. The osteosarcoma samples were chosen as the primary control tissue to avoid gene expression artifacts introduced by cell culturing. However, as the Wnt signaling pathway is known to be affected also in the control group of osteosarcomas [11], we compared the gene expression levels also between osteoblastoma and two different types of cultured cells (MSC and MSC differentiated into osteoblasts). We could confirm that the four genes mentioned above showed high expression levels in osteoblastoma, regardless of the reference group (Figure 2).

### Sanger sequencing did not identify any mutations in candidate targets

The coding regions of the genes *MN1, ZNRF3, KREMEN1* and *NF2* were sequenced in case 2 because this case had showed heterozygous deletions of these genes at SNP array analysis. No mutation was detected (data not shown). Exon 1 of *ZNRF3* and *KREMEN1*, respectively, were not successfully amplified and were thus not evaluated. The lack of amplified material from parts of *ZNRF3* and *KREMEN1* could be due to technical failure and was not interpreted as acquired mutations. 

## Discussion

The genetic mechanisms underlying osteoblastoma development are largely unknown and no obvious genetic difference between conventional and so-called aggressive tumors has been identified [5]. Cytogenetic information is available from six published osteoblastomas and complex rearrangements are detected in some of them whereas others display simple karyotypes with few changes, irrespective of whether the tumors are termed conventional or aggressive (Table 3). In the present study, we found few or no genomic changes in nine conventional osteoblastomas while the two aggressive tumors displayed heavily rearranged genomes. The most common finding was deletion of whole or parts of the long arm of chromosome 22, which was detected by SNP array and/or cytogenetic analyses in three of the cases. Loss of chromosome 22 and rearrangement of 22q12, respectively, were reported in one case each of the previously published osteoblastomas. Furthermore, it could be noted that deletions affecting 22q13 has been described in two of three reported karyotypes from osteoid osteomas [12]. Available data thus indicate that a candidate target gene for osteoblastoma development may reside in the long arm of chromosome 22. In support of this, one of the aggressive tumors investigated here displayed homozygous deletions of three neighboring regions in 22q12. In total, ten genes were affected by the deletions and four of them may be particularly interesting due to their involvement in osteogenesis and/or tumorigenesis; *ZNRF3, KREMEN1, MN1*, and *NF2*. 

**Table 3:** Clinical and cytogenetic features of previously published osteoblastomas.

**Case^a^**	**Age/Sex**	**Location^b^**	**Diagnosis^c^**	**Karyotype**
5004:1	7/M	Vertebra	OB	46,XY,+der(15)t(15;20)(p11;p11),der(17)t(17;20)(p11-12;q11),-20
7676:8	34/M	Os ilium	Aggr OB	52,Y,t(X;11)(q22;p14),+2,del(5)(q22),der(6;8)(p10;q10),+del(9)(q31q33),add(12)(q24),-13,add(13)(p11),add(14)(p?),+16,add(18)(p11),+19,+add(19)(p13),-21,+3mar
8024:16	62/F	N/A	OB	39-40,XX,der(3;8)dic(3;8)(p25;p11)ins(3;?)(p25;?),der(4)t(4;?22)(p15;q12),r(7),-9,der(11)t(11;12)(p13;q12),-12,-14,add(19)(p13),dic(19;22)(q11;p13),-22/39-40,idem,add(19)(p13)
8416:1	14/F	Mandible	LCET OB	46,XX,del(1)(q42),t(1;5;17;22)(p32-33;p13;q21;q12)
13047:1	23/F	Femur	OB	46,XX,t(1;2;14)(q42;q13;q24)
13302:1	12/F	Femur	Aggr OB	46,XX,t(4;7;14)(q23-25;q31;q31)

a Previously published karyotypes are indicated by their reference and case numbers in The Mitelman Database of Chromosome Aberrations and Gene Fusions in Cancer (http://cgap.nci.nih.gov/Chromosomes/Mitelman).

b N/A = not available.

c OB = osteoblastoma; Aggr OB = aggressive osteoblastoma; LCET OB = large cell, epithelioid, telangiectatic OB.

*ZNRF3* and *KREMEN1* are negative regulators of Wnt signaling transduction [13,14]. Wnt normally acts through different pathways to regulate cell proliferation, cell polarity and cell fate during embryogenesis and adult tissue homeostasis [15]. The different Wnt signaling pathways include the canonical and the non-canonical pathways, the former is also known as the beta-catenin-dependent pathway. In this pathway, cytoplasmic beta-catenin is constantly phosphorylated leading to ubiquitination and degradation when Wnt is absent [15]. In contrast, when Wnt protein is present it assembles its receptors - the Frizzled family of receptors and various co-receptors including the LDL receptor-related proteins 5 and 6 (LRP5/6) - which in turn prevents phosphorylation and degradation of beta-catenin. Stabilized beta-catenin will accumulate and translocate to the nucleus to form complexes with transcription factors and activate Wnt target gene expression. The Wnt/beta-catenin pathway regulates, among other things, bone mass and aberrations in this pathway has been found in e.g. osteodegenerative conditions and osteosarcoma [11,15]. To adjust and restrict Wnt signaling activity there are several negative regulators of this pathway. Z*NRF3* encodes a cell-surface transmembrane ubiquitin ligase which reduces Wnt signals by promoting degradation of Frizzled and LRP6 receptors [13,16]. In absence of ZNRF3, membrane levels of Wnt receptors increase and this enhances Wnt signaling through both the canonical and non-canonical pathways [13]. KREMEN1 is a transmembrane receptor that inhibits the Wnt pathway by forming a ternary complex with Dickkopf1 (Dkk1) and LRP5/6 [14]. When assembled, this complex is removed from the plasma membrane by endocytosis, thereby blocking Wnt signaling through LRP5/6. Taken together, both loss of *ZNRF3* and *KREMEN1* would theoretically result in increased accumulation of beta-catenin that will translocate to the nucleus and activate Wnt target gene expression. In line with this, loss of *ZNRF3* has been shown to result in accumulation of beta-catenin and loss of *KREMEN1* has been implicated in increased bone formation [13,17,18]. Here, we hypothesized that if loss of genes such as *ZNRF3* and *KREMEN1* and subsequent activation of beta-catenin is important for osteoblastoma development we would find high expression levels of genes activated by Wnt/beta-catenin in these tumors. Indeed, Wnt/beta-catenin target genes involved in bone metabolism, such as *BMP2, BMP4, PTGS2* and *MMP16*, showed high expression levels in osteoblastoma [19-25]. In line with this, both membranous and nuclear beta-catenin has been found to be strongly expressed in osteoblastoma [11]. Furthermore, constitutional *APC* mutations leading to accumulation of beta-catenin is associated with the development of osteoma, a benign bone lesion that shares some morphological features with osteoid osteoma and osteoblastoma [26,27].

*ZNRF3* and *KREMEN1* may thus be potential target genes of one of the homozygously deleted regions. Possible targets of the two adjacent, homozygously deleted regions may be *MN1* and *NF2*. *MN1* encodes a transcription regulator and was originally identified as a gene disrupted in meningioma and as part of a fusion gene in leukemia. MN1 has been shown to be involved in osteoblast proliferation and differentiation [28]. More specifically, osteoblasts derived from *MN1* knock-out mice are defective in osteoblast proliferation, migration, differentiation and mineralization. The skeletal defects of the *MN1* knock-out seem to primarily affect cranial skeletal elements while long bones of the appendicular skeleton appear to develop normally. Mutations in *NF2* are associated with the autosomal dominant disorder neurofibromatosis type 2 [29]. Affected patients primarily develop tumours of the nervous system, including schwannomas, meningiomas and ependymomas. *NF2* has not been specifically associated with bone formation. Instead, in patients with craniofacial dysmorphism and concomitant deletions affecting *NF2*, the deletions have been shown to encompass also the *MN1* gene, supporting the role of *MN1* in human craniofacial development [30]. The protein product of *NF2* is also known to inhibit many signaling pathways at the membrane and in the nucleus, including the Wnt/beta-catenin pathway [29]. 

## Conclusions

Most of the tumors investigated in the present study were conventional osteoblastomas and they showed very few or no acquired genetic aberrations. This was in contrast to the two epithelioid or aggressive osteoblastomas, which both displayed heavily rearranged genomes. Few aberrations were recurrent and the most intriguing findings were three neighboring homozygous deletions affecting 22q12 in one of the aggressive cases. Loss or rearrangement of the long arm of chromosome 22 was found in two additional cases investigated here, as well as two previously published osteoblastomas and two reported osteoid osteomas. These aberrations are thus not specific for the aggressive subtype of osteoblastoma but rather seem to affect osteoblastoma regardless of subtype as well as the closely related tumor type osteoid osteoma. The pathogenetic importance of these aberrations is not known but four of the homozygously deleted genes are involved in osteogenesis and/or tumorigenesis. Three of them - *ZNRF3*, *KREMEN1* and *NF2* - are inhibitors of the Wnt/beta-catenin pathway. Losses of these genes and subsequent accumulation of beta-catenin are in line with the high protein expression of beta-catenin previously detected in osteoblastoma.

## Supporting Information

Table S1 **Primers and PCR protocols for mutation screening of MN1*,* NF2*, KREMEN1* and *ZNRF3*.**
(PDF)

Table S2 **Genes with a high expression value in osteoblastoma compared to osteosarcoma.** Significance level was set to p<0.001 and FDR (q-value)<0.01.
(XLSX)

Table S3 **Genes with a low expression value in osteoblastoma compared to osteosarcoma.** Significance level was set to p<0.001 and FDR (q-value)<0.01.
(XLSX)
