# Supplementary material for: Recurrent Chromosome 22 Deletions in Osteoblastoma Affect Inhibitors of the Wnt/Beta-Catenin Signaling Pathway
Source: PLoS One. 2013 Nov 13;8(11):e80725. doi: 10.1371/journal.pone.0080725 (PMC3827481; doi:10.1371/journal.pone.0080725)
Supplement: Table S1 — Primers and PCR protocols for mutation screening of MN1, NF2, KREMEN1 and ZNRF3. (PDF) [file pone.0080725.s001.pdf]

**Table S1. Primers and PCR protocols for mutation screening of *MN1, NF2, KREMEN1* and *ZNRF3.***

***MN1*** (Accession No. NM_002430)

| **Designation** | **Sequence** | **Exon** |
|---|---|---|
| MN1-1514F | 5’-GGCTGTCATGCCCTATTGAT | Exon 1 |
| MN1-1882R | 5’-CTGGTGGGGATGATGACTTC | Exon 1 |
| MN1-5000F | 5’-GAGATCCACCCCCTGGAG | Exon 1 |
| MN1-5375R | 5’-GTCACCCGGGAAGTGAGAG | Exon 1 |
| MN1-10120F | 5’-GGCAGGACCAGGTGAAATTA | Exon 2 |
| MN1-10440R | 5’-CTCCCCAGACATTTGATCCT | Exon 2 |
| MN1-50900F | 5’-GGACGGTGCCTGTCTTGTAT | Exon 3 |
| MN1-51296R | 5’-TCAGCAATAGTGGCCCTTTC | Exon 3 |
| MN1-51257F | 5’-CCCCACCCCTCTGTTAATTT | Exon 3 |
| MN1-51642R | 5’-TAAAAGGGGAGGGGGTAAGA | Exon 3 |
| MN1-51576F | 5’-TTTCATAATAAGACAAGAGTTGCTTTC | Exon 3 |
| MN1-51923R | 5’-GCTTCCAGCAGGAGAAGAGA | Exon 3 |
| MN1-51856F | 5’-TTCCTGCAAACACAGTGCTC | Exon 3 |
| MN1-52255R | 5’-ATGGGAGAGGTGGAGGAGAT | Exon 3 |
| MN1-52205F | 5’-CATCCCAGGGAAGAAGGAG | Exon 3 |
| MN1-52608R | 5’-GCCATAAATGGCTTTTGCAG | Exon 3 |
| MN1-52569F | 5’-ACTTTGGGTGCTGCTGATG | Exon 3 |
| MN1-52975R | 5’-GAGAACCGCAGACTCAGACC | Exon 3 |
| MN1-52931F | 5’-CCTTCTGCCGGTCCTTTT | Exon 3 |
| MN1-53303R | 5’-AACGCTGGGAAAGTCAAAGA | Exon 3 |
| MN1-53259F | 5’-CGGGGAGCAAAGTGTCTTAAC | Exon 3 |
| MN1-53613R | 5’-GGATTGCCTAACACACAGCA | Exon 3 |
| MN1-53564F | 5’-TTGCACATGTAAAATATGAAAACTT | Exon 3 |
| MN1-53931R | 5’-TCCAAACCTACACCCCTACG | Exon 3 |

PCR reactions for exons 2 and 3 were performed in a 50 µl total reaction volume containing 1x PCR buffer, 1 mM dNTPs, 1.25 mM $MgCl_2$, 0.5 µM of each of the forward and reverse primers, 1 U Platinum Taq DNA Polymerase (Life Technologies, Foster City, USA) and 100 ng genomic DNA template. After denaturation for 2 min at 94 C, 30 cycles of 30 s at 94 C, 30 sec at 58 C and 1 min at 72 C were run, followed by a final extension for 3 min at 72 C. Amplified fragments were purified from agarose gels and directly sequenced using the Big Dye v1.1 cycle sequencing kit (Life Technologies) on an ABI-3100 Avant genetic analyzer (Life Technologies).
MN1 exon 1 is previously known to be difficult to amplify. Therefore, a special kit for challenging amplicons, KAPA2G Robust Hot Start (Kapa Biosystems,Woburn, M.A., U.S.), was used. PCR reactions for were performed in a 25 µl total reaction volume containing 50% KAPA Mix, 0.5 µM of each of the forward and reverse primers, 5% DMSO and 100 ng genomic DNA template. After denaturation for 3 min at 95 C, 35 cycles of 15 s at 95 C, 15 sec at 56 C and 1 min at 72 C were run, followed by a final extension for 2 min at 72 C. 1 µl PCR-product was used as template for a second PCR with the same protocol and program as the first PCR. Amplified fragments were purified from agarose gels and directly sequenced using the Big Dye v1.1 cycle sequencing kit (Life Technologies) on an ABI-3100 Avant genetic analyzer (Life Technologies).

***NF2*** (Primers are according to Jacoby LB, MacCollin M, Louis DN, Mohney T, Rubio MP, et al. (1994) Exon scanning for mutation of the *NF2* gene in schwannomas. Hum Mol Genet 3: 413-419.)

| **Designation** | **Sequence** | **Exon** |
|---|---|---|
| NF2-exon1F | 5’-GCTAAAGGGCTCAGAGTGCAG | Exon 1 |
| NF2-exon1R | 5’-GAGAACCTCTCGAGCTTCCAC | Exon 1 |
| NF2-exon2F | 5’-AGTGCAGAGAAAAGGTTTTATTAATGAT | Exon 2 |
| NF2-exon2R | 5’-TGGAAAGCTCACGTCAGCC | Exon 2 |
| NF2-exon3F | 5’-GCTTCTTTGAGGGTAGCACA | Exon 3 |
| NF2-exon3R | 5’-GGTCAACTCTGAGGCCAACT | Exon 3 |
| NF2-exon4F | 5’-CCTCACTTCCACTCACAGAG | Exon 4 |
| NF2-exon4R | 5’-CCCATGACCCAAATTAACGC | Exon 4 |
| NF2-exon5F | 5’-TGGCAGTTATCTTTAGAATCTC | Exon 5 |
| NF2-exon5R | 5’-TTAGACCACATATCTGCTATG | Exon 5 |
| NF2-exon6F | 5’-CATGTGTAGGTTTTTTATTTTGC | Exon 6 |
| NF2-exon6R | 5’-GCCCATAAAGGAATGTAAACC | Exon 6 |
| NF2-exon7F | 5’-CCATCTCACTTAGCTCCAATG | Exon 7 |
| NF2-exon7R | 5’-CTCACTCAGTCTCTGTCTAC | Exon 7 |
| NF2-exon8F | 5’-GAAGGTTGAATAAAATTTTGAGCCTC | Exon 8 |

| | | |
|---|---|---|
| NF2-exon8R | 5’-GACAGGGAAAGATCTGCTGGACC | Exon 8 |
| NF2-exon9F | 5’-GACTTGGTGCTCCTAATTCCC | Exon 9 |
| NF2-exon9R | 5’-CCATTATCAGTAATGAAAACCAGG | Exon 9 |
| NF2-exon10F | 5’-TGCTACCTGCAAGAGCTCAA | Exon 10 |
| NF2-exon10R | 5’-CTGACCACACAGTGACATC | Exon 10 |
| NF2-exon11F | 5’-TCTTTGGGCCCTTGTGGCAC | Exon 11 |
| NF2-exon11R | 5’-CAGGAGACCAAGCTCCAGAA | Exon 11 |
| NF2-exon12AF | 5’-TTCAGCTAAGAGCACTGTGC | Exon 12A |
| NF2-exon12AR | 5’-CGCTGCATTTCCTGCTCAG | Exon 12A |
| NF2-exon12BF | 5’-GCTGAAAAGGCCCAGATCA | Exon 12B |
| NF2-exon12BR | 5’-CTTGAGGACAACTGCTGTAG | Exon 12B |
| NF2-exon13F | 5’-GGTGTCTTTTCCTGCTACCT | Exon 13 |
| NF2-exon13R | 5’-GGGAGGAAAGAGAACATCAC | Exon 13 |
| NF2-exon14F | 5’-TGTGCCCATTGCCTCTGTG | Exon 14 |
| NF2-exon14R | 5’-AGGGCACAGGGGGCTACA | Exon 14 |
| NF2-exon15F | 5’-TCTGCCCAAGCCCTGATGC | Exon 15 |
| NF2-exon15R | 5’-TGGTCCTGATCAGCAAAATAC | Exon 15 |
| NF2-exon16F | 5’-GGCATTGTTGATATCACAGGG | Exon 16 |
| NF2-exon16R | 5’-GGCAGCACCATCACCACATA | Exon 16 |
| NF2-exon17F | 5’-CTCTCAGCTTCTTCTCTGCT | Exon 17 |
| NF2-exon17R | 5’-CCAGCCAGCTCCCTATGGATG | Exon 17 |

PCR reactions were performed in a 50 µl total reaction volume containing 1x PCR buffer, 1 mM dNTPs, 1.25 mM $MgCl_2$, 0.5 µM of each of the forward and reverse primers, 1 U Platinum Taq DNA Polymerase (Life Technologies, Foster City, USA) and 100 ng genomic DNA template. After denaturation for 2 min at 94 C, 30-35 cycles of 30 s at 94 C, 30 sec at 58-60 C and 1 min at 72 C were run, followed by a final extension for 3 min at 72 C. Amplified fragments were purified from agarose gels and directly sequenced using the Big Dye v1.1 cycle sequencing kit (Life Technologies) on an ABI-3100 Avant genetic analyzer (Life Technologies).

***KREMEN1*** (ENSG00000183762)

| **Designation** | **Sequence** | **Exon** |
|---|---|---|
| KREMEN1-7F | 5’-CTGCTGATCCTACCCTTGGA | Exon 1 |
| KREMEN1-468R | 5’-GGGGTCGCTCACACTCAC | Exon 1 |
| KREMEN1-1031F | 5’-CCATGAATGTTGCCAGAAAA | Exon 2 |
| KREMEN1-1312R | 5’-TGAAAGGGGTTGGAAGAAGA | Exon 2 |
| KREMEN1-1885F | 5’-TGTGTCCTTTTCCCCAACAG | Exon 3 |
| KREMEN1-2032R | 5’-TCCTGTAAACCATCACATTGG | Exon 3 |
| KREMEN1-2643F | 5’-TGTTGCGAAATTCTTTTGTTTTT | Exon 4 |
| KREMEN1-2825R | 5’-ACACAGCCACAGAGTCATCA | Exon 4 |
| KREMEN1-3424F | 5’-GTTGAGGCTGATTGCTTGCT | Exon 5 |
| KREMEN1-3701R | 5’-GATGGGCACAGGGCATACT | Exon 5 |
| KREMEN1-4298F | 5’-GGCGTGGTGTCTTTTTCTCT | Exon 6 |
| KREMEN1-4680R | 5’-CCAAGGAGGCAAAGATGTC | Exon 6 |
| KREMEN1-5568F | 5’-CATAGCCCTGTCCCCAGTTA | Exon 7 |
| KREMEN1-5957R | 5’-CACCCAAGTGCTTTTGTCTTT | Exon 7 |
| KREMEN1-7207F | 5’-CTCCTTTCGAAAACCAACCA | Exon 8 |
| KREMEN1-7372R | 5’-GGGCAGCTCCCTCTTGTACT | Exon 8 |
| KREMEN1-8774F | 5’-CCCCACCCCATTTCATAGAT | Exon 9 |
| KREMEN1-9007R | 5’-ACCACAGGAGAGGTTGACCA | Exon 9 |
| KREMEN1-9613F | 5’-CGTGCCTAGGGCTGTACTTT | Exon 10 |
| KREMEN1-9774R | 5’-AAATTGCCACCCAGATATGC | Exon 10 |

PCR reactions were performed in a 50 µl total reaction volume containing 1x PCR buffer, 1 mM dNTPs, 1.25 mM $MgCl_2$, 0.5 µM of each of the forward and reverse primers, 1 U Platinum Taq DNA Polymerase (Life Technologies, Foster City, USA) and 100 ng genomic DNA template. After denaturation for 2 min at 94 C, 30 cycles of 30 s at 94 C, 30 sec at 57-58 C and 1 min at 72 C were run, followed by a final extension for 3 min at 72 C. Amplified fragments were purified from agarose gels and directly sequenced using the Big Dye v1.1 cycle sequencing kit (Life Technologies) on an ABI-3100 Avant genetic analyzer (Life Technologies).

***ZNRF3*** (ENSG00000183579)

| Designation | Sequence | Exon |
|---|---|---|
| ZNRF3-223F | 5’-AGCCTGCGACCCACAAAG | Exon 1 |
| ZNRF3-593R | 5’-CGGTGGTGTAGGTGGTGTAA | Exon 1 |
| ZNRF3-570F | 5’-GCGATTACACCACCTACACCA | Exon 1 |
| ZNRF3-1060R | 5’-GGTTCACAGGAGCTCTAGCC | Exon 1 |
| ZNRF3-1448F | 5’-CGTTACTTTTGGCTATAGCATCTG | Exon 2 |
| ZNRF3-1734R | 5’-TGCTGAAATGGAAGAGTGGA | Exon 2 |
| ZNRF3-2233F | 5’-GATTGCCAAGGCCAACTTT | Exon 3 |
| ZNRF3-2453R | 5’-GGGACAAGCCAAGCTCACTA | Exon 3 |
| ZNRF3-3069F | 5’-AAGCGCACCTTTTAACCAGA | Exon 4 |
| ZNRF3-3269R | 5’-GAGCCTGTGCCAAATGGT | Exon 4 |
| ZNRF3-4545F | 5’-GCTGGAGAGAGGCTGTGACT | Exon 5 |
| ZNRF3-4734R | 5’-GGTTACTGCTCACGGGCTAC | Exon 5 |
| ZNRF3-6486F | 5’-CGTACTGGACCCTCACACAG | Exon 6 |
| ZNRF3-6744R | 5’-CTAGGTCTTGGGCACCTCTG | Exon 6 |
| ZNRF3-8167F | 5’-GCAGACTTGTGTCCCCTCTC | Exon 7 |
| ZNRF3-8394R | 5’-ACGGTGGTTCTGAGTGTTCC | Exon 7 |
| ZNRF3-8963F | 5’-TATGCTCAGCCCTGCCTACT | Exon 8 |
| ZNRF3-9984F | 5’-TGCAGCCTGGAGATGAACTA | Exon 8 |
| ZNRF3-10008R | 5’-AGGGAGGAGTTGCTGCTGTA | Exon 8 |
| ZNRF3-10816R | 5’-AGACAAGACGACCCTGATGG | Exon 8 |
| ZNRF3-13355F | 5’-CCTCCCCTGATGATTGTTCT | Exon 9 |
| ZNRF3-13472R | 5’-CCATACAGTTCCCAATTTCCA | Exon 9 |

PCR reactions were performed in a 50 µl total reaction volume containing 1x PCR buffer, 1 mM dNTPs, 1.25 mM $MgCl_2$, 0.5 µM of each of the forward and reverse primers, 1 U Platinum Taq DNA Polymerase (Life Technologies, Foster City, USA) and 100 ng genomic DNA template. After denaturation for 2 min at 94 C, 30 cycles of 30 s at 94 C, 30 sec at 58 C and 2 min at 72 C were run, followed by a final extension for 3 min

at 72 C.  Amplified fragments were purified from agarose gels and directly sequenced using the Big Dye v1.1 cycle sequencing kit (Life Technologies) on an ABI-3100 Avant genetic analyzer (Life Technologies).
